# Electrophysiological differences of randomized deep sedation with dexmedetomidine versus propofol

**DOI:** 10.1186/s12871-024-02647-x

**Published:** 2024-07-31

**Authors:** Helge Servatius, Thomas Kueffer, Gabor Erdoes, Jens Seiler, Hildegard Tanner, Fabian Noti, Andreas Haeberlin, Antonio Madaffari, Mattia Branca, Sophie Dütschler, Lorenz Theiler, Tobias Reichlin, Laurent Roten

**Affiliations:** 1 Department of Cardiology, Inselspital, Bern University Hospital, University of Bern, Bern, Switzerland; 2 Department of Anaesthesiology and Pain Medicine, Inselspital, Bern University Hospital, University of Bern, Bern, Switzerland; 3 CTU Bern, University of Bern, Bern, Switzerland; 4 Department of Anaesthesiology, Kantonsspital Aarau, Aarau, Switzerland

**Keywords:** Sedation, Catheter ablation, Atrial fibrillation, Propofol, Dexmedetomidine

## Abstract

**Background:**

Dexmedetomidine and propofol are common sedatives in intensive care units and for interventional procedures. Both may compromise sinus node function and atrioventricular conduction.

The objective of this prospective, randomized study is to compare the effect of dexmedetomidine with propofol on sinus node function and atrioventricular conduction.

**Methods:**

In a tertiary care center in Switzerland we included from September 2019 to October 2020 160 patients (65 ± 11 years old; 32% female) undergoing first ablation for atrial fibrillation by cryoballoon ablation or by radiofrequency ablation.

Patients were randomly assigned to deep sedation with dexmedetomidine (DEX group) versus propofol (PRO group). A standard electrophysiological study was performed after pulmonary vein isolation with the patients still deeply sedated and hemodynamically stable.

**Results:**

Eighty patients each were randomized to the DEX and PRO group. DEX group patients had higher baseline sinus cycle length (1022 vs. 1138 ms; *p* = 0.003) and longer sinus node recovery time (SNRT400; 1597 vs. 1412 ms; *p* = 0.042). However, both corrected SNRT and normalized SNRT did not differ. DEX group patients had longer PR interval (207 vs. 186 ms; *p* = 0.002) and AH interval (111 vs. 95 ms, *p* = 0.008), longer Wenckebach cycle length of the atrioventricular node (512 vs. 456 ms; *p* = 0.005), and longer atrioventricular node effective refractory period (390 vs. 344 ms; *p* = 0.009). QRS width and HV interval were not different. An arrhythmia, mainly atrial fibrillation, was induced in 33 patients during the electrophysiological study, without differences among groups (20% vs. 15%, *p* = 0.533).

**Conclusions:**

Dexmedetomidine has a more pronounced slowing effect on sinus rate and suprahissian AV conduction than propofol, but not on infrahissian AV conduction and ventricular repolarization. These differences need to be taken into account when using these sedatives.

**Trial registration:**

ClinicalTrials.gov number NCT03844841, 19/02/2019

## Introduction

With the evolution of medical technologies, medicine has witnessed a shift from traditional, surgical interventions to interventional procedures. The latter are often performed on an outpatient basis and may involve complex and long-lasting interventions. General anesthesia is usually avoided during such procedures and mild to deep sedation is applied instead. Depending on local policy, sedation may be performed by the interventionalists themselves without anesthesiologist support. Traditionally, benzodiazepines (such as midazolam) and opioids have been used for sedation. However, to optimize patient comfort and procedural safety, propofol and dexmedetomidine are frequently employed nowadays. These drugs are used for interventional procedures in various settings, including cardiology, gastroenterology, pulmonology, neurosurgery, ophthalmology, intensive care and many more.[1–10].

Besides effects on hemodynamics and ventilation, both drugs may also influence cardiac chronotropy and dromotropy. Dexmedetomidine, which is an alpha-2 adrenergic agonist, does in particular affect heart rate and atrioventricular nodal conduction and may induce sinus arrest or complete heart block in susceptible patients. Bradycardia on the other side has also been described after propofol administration. No study to date has directly compared the electrophysiological effects of these two drugs in a randomized controlled trial.

## Materials and methods

In this study, patients with atrial fibrillation undergoing pulmonary vein isolation were randomized to sedation with propofol (PRO group) versus dexmedetomidine (DEX group). The primary study outcome was a composite endpoint of inefficient sedation, respiratory depression and hemodynamic changes. The methods and main results of the study have been published elsewhere.[11] In brief, a total of 160 patients undergoing first pulmonary vein isolation for atrial fibrillation by cryoballoon ablation or by radiofrequency ablation were randomized 1:1 to procedural sedation with dexmedetomidine versus propofol.

Patient characteristics were collected on patient inclusion and a standard 12-lead ECG was performed the day before the procedure. On the 12-lead ECG, RR, PR and QT intervals were measured in leads II or V5. These measurements were repeated on the 12-lead ECG during the electrophysiological study. The Bazett formula was used to correct the QT interval for heart rate (QTc).

### Ethics

Ethical approval for this study (N° 2018–02128) was provided by the Ethical Committee of the Kanton of Bern (Kantonale Ethikkommission, Murtenstrasse 31, Hörsaaltrakt Pathologie, Eingang 43A, Büro H372, 3010 Bern; Chairperson Prof Robert Greif) on 12 February 2019. All patients provided written informed consent to participate.

### Procedural sedation

Upon arrival of the patient at the operating room, 50 µg fentanyl and 1 mg of midazolam were given. Patients in the dexmedetomidine arm additionally received 4 mg of ondansetron. After five minutes, sedation with propofol or dexmedetomidine was started if the patient was stable.

For propofol sedation we used a target-controlled infusion (TCI) pump using the Schnider pharmacokinetic model with an effect-site propofol concentration initially set to 1.2 µg/ml, unless the patient was already sedated by the initial fentanyl and midazolam dose, in which case an effect-site propofol concentration of 0.8–1.0 µg/ml was chosen. During the procedure, the effect-site propofol concentration was adjusted stepwise (by 0.2 µg/ml) to reach a target score of 3 on the “Modified Observer’s Assessment of Alertness/Sedation” (MOAA/S) scale.

For dexmedetomidine sedation we infused a dexmedetomidine loading dose of 0.8 µg/kg over 3 min via a perfusor pump, which was automatically continued at a maintenance dose of 0.8 µg/kg/h after loading. If the patient was already sedated by the premedication, no dexmedetomidine loading dose was administered and dexmedetomidine initiated at a maintenance dose of 0.4–0.8 µg/kg/h. During the procedure, the dexmedetomidine maintenance dose was adjusted stepwise (using steps of 0.2 µg/ml) to reach a target score of 3 on the MOAA/S scale.

All patients in both arms received an additional bolus of fentanyl (20–50 µg) just before beginning of ablation, and additional fentanyl was administered bolus-wise (10–30 µg) at the discretion of the treating electrophysiologist, as necessary. Addition of 1–2 mg of midazolam during the procedure was allowed in case of anxious or agitated patients. Vasoactive agents were not allowed, since they would have altered the endpoints of the main study. If patients were in atrial fibrillation we performed electrical cardioversion after deepening sedation to a target score of 2 on the MOAA/S scale. To achieve this, either the effect-site propofol concentration was temporarily increased in PRO group patients, or propofol boluses of 20 mg were added stepwise in DEX group patients. The electrophysiological study was performed during the waiting period after isolation of the pulmonary veins with the patients in sinus rhythm and hemodynamically stable. Deep sedation with propofol or dexmedetomidine was continued unchanged during the electrophysiological study.

### Electrophysiological study

A decapolar catheter was placed at the His for measurements of atrial—His- (AH) and His – ventricular (HV) intervals. To assess the Wenckebach cycle length of the atrioventricular (AV) node, we paced the atrium with decreasing cycle length (in steps of 10 ms) until we observed loss of 1:1 AV conduction. To measure the effective refractory period of the atrium and AV node we delivered a drive train (S1) of six paced beats with a cycle length of 600 ms followed by a premature, paced beat (S2) at a programmed coupling interval. The coupling interval of S2 was decreased in steps of 10 ms for each consecutive drive train until loss of atrial activation or AV conduction occurred, respectively. The effective refractory period of the atrium and AV node was the longest coupling interval of S2, which failed to elicit atrial activation or AV node conduction, respectively. To determine the sinus node recovery time (SNRT) we paced the atrium for 30 s at cycle lengths of 600, 500 and 400 ms, and then stopped pacing abruptly. The SNRT was the pause induced by this overdrive suppression until the first sinus beat (interval following the cessation of pacing). If an ectopic beat occurred after cessation of pacing, the test was repeated. To calculate the corrected SNRT (cSNRT), we subtracted the sinus cycle length (SCL) from the SNRT. The SCL was measured just before pacing the atrium for 30 s at the respective cycle lengths. To calculate the normalized SNRT (nSNRT) we divided the SNRT by the SCL. Finally, we assessed whether atrial fibrillation or any other supraventricular tachycardia occurred during the electrophysiological study.

### Statistical analyses

Continuous variables are expressed as means with standard deviations or medians with interquartile ranges (IQR), and categorical variables as frequencies with percentages. Continuous variables were compared using the Mann–Whitney U test or t-test in case of two-group comparison. Differences in proportions were tested with Pearson’s χ2 test or Fisher’s exact test, as appropriate. The relationship between the continuous variables (RR-, PR-, and AH-interval as well as Wenckebach CL of the AV node) and selected demographic and clinical variables were computed using a linear regression model accounting for robust standard errors. To account for possible associations among variables, a backward selection considering the most predictive variables in the univariable results (with selection of p-value lower than 0.2) was performed. Consequently, multivariable models were estimated to measure the influence of the adjusted effects considering only the most informative covariates, where the AIC was applied to select the most predictive variables. All analyses were performed using Stata 17.0 (StataCorp. Stata Statistical Software: Release 17. College Station, TX: StataCorp LLC).

## Results

### Patient characteristics

In total, 160 patients (mean age 65 years; 32% females) were enrolled in the study from September 2019 to October 2020. Of these, 80 patients (50%) received dexmedetomidine and 80 (50%) propofol sedation regimen. Patient characteristics are shown in Table 1. In 79 patients, point by point radiofrequency ablation was used and in 81 patients, cryoballoon ablation was used.

**Table 1:** Patient characteristics

	**All** ***N* = 160**	**DEX group** ***N* = 80**	**PRO group** ***N* = 80**	***P* value**
Age, years	65 ± 11	66 ± 10	64 ± 12	0.446
Gender, female	51 (32%)	28 (35%)	23 (29%)	0.396
Body mass index, kg/m^2^	27 ± 4	26 ± 4	27 ± 4	0.074
Arterial hypertension	86 (54%)	44 (55%)	42 (53%)	0.751
Diabetes mellitus	12 (8%)	5 (6%)	7 (9%)	0.548
Coronary artery disease	16 (10%)	10 (13%)	6 (8%)	0.292
History of stroke/embolism	14 (9%)	7 (9%)	7 (9%)	1.000
Peripheral artery disease	14 (9%)	6 (8%)	8 (10%)	0.576
History of congestive heart failure	19 (12%)	12 (15%)	7 (9%)	0.222
CHA_2_DS_2_Vasc score	2 (1; 3)	2 (1; 3)	2 (1; 3)	0.608
Obstructive sleep apnea	14 (9%)	6 (8%)	8 (10%)	0.576
Pacemaker or ICD	5 (3%)	4 (5%)	1 (1%)	0.173
Paroxysmal atrial fibrillation	115 (72%)	59 (74%)	56 (70%)	0.598
Previous cardioversion	51 (32%)	27 (34%)	24 (30%)	0.611
LVEF, %	58 ± 8	57 ± 9	59 ± 7	0.140
LAVI ml/m^2^	38 ± 13	40 ± 14	36 ± 12	0.083
**Drugs**				
Betablocker	99 (63%)	47 (59%)	52 (67%)	0.304
Calcium channel blocker	1 (1%)	1 (1%)	-	1.000
Digoxin	1 (1%)	1 (1%)	-	1.000
Flecainid/propafenon	25 (16%)	11 (14%)	14 (18%)	0.470
Amiodarone	2 (1%)	-	2 (3%)	0.242
Sotalol	7 (4%)	5 (6%)	2 (3%)	0.443
**ECG**				
RR interval, ms^a^	178 ± 37	184 ± 41	171 ± 31	0.060
PR, ms*	1014 ± 208	1011 ± 231	1017 ± 181	0.875
QRS, ms	100 ± 16	102 ± 18	99 ± 14	0.252
QT, ms	418 ± 51	423 ± 54	413 ± 47	0.223
QTc (Bazett formula), ms	437 ± 29	442 ± 29	432 ± 28	0.042

*BPM* beats per minute, *ICD* internal cardioverter defibrillator, *LAVI* left atrial volume index, *LVEF* left ventricular ejection fraction

Data are provided as mean ± standard deviation, as median with interquartile range (1st; 3rd) or frequencies with percentages

^a^only for patients in sinus rhythm

### Sedation characteristics

A mean of 231 ± 111 mcg dexmedetomidine was administered in DEX group patients, and a mean of 657 ± 356 mg of propofol in PRO group patients. Table 2. gives all details regarding sedatives administered during the procedure for both groups. Mean and minimal MOAA/S score were not different among groups. At the end of the procedure, when the electrophysiological study was performed, the majority of all patients had a MOAA/S score ≤ 3.[11].

**Table 2.** Sedatives use and procedural characteristics

	**DEX group** ***N* = 80**	**PRO group** ***N* = 80**	***P* value**
Dexmedetomidine, mcg	231 ± 111	-	-
Propofol, mg	-	657 ± 356	-
Fentanyl, mcg	134 ± 52	151 ± 53	0.044
Midazolam, mg	1 (range 1–3)	1 (range 1–3)	0.890
Electrical cardioversion	37 (46%)	33 (41%)	0.524
Additional propofol for cardioversion	25 (68%)	-	-
Additional propofol dose for cardioversion, mg	20 (20; 40)	-	-
Mean MOAA/S score (median)	3 (2; 3)	2 (2; 3)	0.155
Minimal MOAA/S score (median)	2 (2; 2)	2 (2; 2)	0.146
Procedure duration, min	129 ± 57	126 ± 61	0.796

Data are provided as mean ± standard deviation, as median with interquartile range (1st; 3rd), as median with range or frequencies with percentages

### Sinus Node Function according to the type of sedation used

During the electrophysiological study, mean sinus CL was higher in DEX group compared to PRO group patients (1138 vs. 1022 ms; *p* = 0.003; Table 3). In DEX group patients, absolute SNRT was longer when pacing at cycle lengths of 600 ms and 400 ms and with a trend towards a longer SNRT when pacing at cycle length 500 ms. Both cSNRT and nSNRT were not different among groups for all three pacing cycle lengths (Table 3). There was a trend towards a longer atrial ERP in DEX group patients (270 vs. 255 ms; *p* = 0.051). The number of atrial arrhythmias induced during the electrophysiological study was not different among groups (26% vs. 17%; *p* = 0.174), and atrial fibrillation was the most frequent arrhythmia induced in both groups (16 in DEX group [20%] vs. 12 in PRO group [15%]; *p* = 0.533).

**Table 3:** Electrophysiological testing

	**All** ***N* = 160**	**DEX group** ***N* = 80**	**PRO group** ***N* = 80**	***P*value**
RR interval, ms	1079 ± 242	1138 ± 251	1022 ± 220	0.003
PR interval, ms	196 ± 41	207 ± 46	186 ± 32	0.002
QRS duration, ms	104 ± 20	106 ± 23	102 ± 18	0.249
QT interval, ms	432 ± 52	447 ± 49	417 ± 52	< 0.001
QTc interval (Bazett formula), ms	420 ± 41	424 ± 42	416 ± 39	0.226
AH interval, ms	103 ± 35	111 ± 40	95 ± 28	0.008
HV interval, ms	44 ± 9	44 ± 8	44 ± 9	0.868
Wenckebach CL of AV node, ms	483 ± 118	512 ± 139	456 ± 88	0.005
ERP of the atrium, ms	262 ± 42	270 ± 47	255 ± 37	0.051
ERP of the AV node, ms	366 ± 104	390 ± 118	344 ± 85	0.009
SNRT @ pacing with CL 600 ms				
Sinus cycle length, ms	1101 ± 234	1153 ± 224	1052 ± 234	0.010
SNRT, ms	1482 ± 388	1581 ± 390	1386 ± 363	0.003
cSNRT, ms	381 ± 331	427 ± 353	336 ± 305	0.106
nSNRT, %	136 ± 30	139 ± 31	134 ± 29	0.266
SNRT @ pacing with CL 500 ms				
Sinus cycle length, ms	1122 ± 257	1193 ± 260	1054 ± 237	0.001
SNRT, ms	1537 ± 577	1635 ± 743	1447 ± 346	0.055
cSNRT, ms	418 ± 497	451 ± 671	387 ± 242	0.452
nSNRT, %	139 ± 41	139 ± 54	138 ± 23	0.901
SNRT @ pacing with CL 400 ms				
Sinus cycle length, ms	1141 ± 264	1206 ± 269	1082 ± 247	0.005
SNRT, ms	1501 ± 519	1597 ± 628	1412 ± 377	0.042
cSNRT, ms	359 ± 405	393 ± 511	328 ± 273	0.364
nSNRT, %	132 ± 33	133 ± 40	132 ± 26	0.842
Arrhythmia induced during EPS	33 (21%)	20 (26%)	13 (17%)	0.174
Atrial fibrillation	28 (18%)	16 (20%)	12 (15%)	0.533
Atrial flutter	3 (2%)	-	3 (4%)	0.245
AV nodal reentry tachycardia	1 (1%)	1 (1%)	-	1.000
Atrial tachycardia	3 (2%)	3 (4%)	-	0.245
Other/undetermined	1 (1%)	1 (1%)	-	1.000

*AV* atrioventricular, *CL* cycle length, *CSNRT* corrected SNRT, *EPS* electrophysiological study, *ERP* effective refractory period, *NSRT* normalized SNRT, *QTc* corrected QT interval, *SNRT* sinus node recovery time

Data are provided as mean ± standard deviation or frequencies with percentages

### AV Node function according to the type of sedation used

**Fig. 1:**
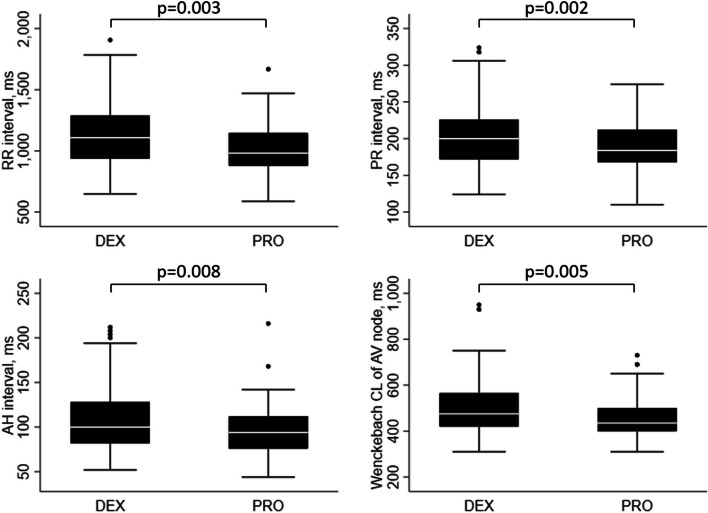
Boxplots showing RR, PR and AH interval and Wenckebach cycle length (CL) of the atrioventricular (AV) node for DEX and PRO group patients. DEX: dexmedetomidine; PRO: propofol

PR interval (207 vs. 186 ms; *p* = 0.002), AH interval (111 vs. 95 ms; *p* = 0.008), the ERP of the AV node (390 vs. 344 ms; *p* = 0.009) and the Wenckebach cycle length of the AV node (512 vs. 456 ms; *p* = 0.005) were all longer in DEX group patients compared to PRO group patients (Table 3 and Fig. 1).

### Venticular De- and repolarization according to the type of sedation used

QRS duration and HV interval were not different among groups. The QT interval was longer (447 vs. 417 ms; *p* < 0.001; Table 3) in DEX group patients. However, the corrected QT interval was not different among groups (424 vs. 416 ms; *p* = 0.226).

### Predictors of sinus node and AV-Node properties

In univariable analysis, the RR interval correlated with DEX group assignment, older age and larger LAVI (Table 4). In multivariable analysis, these variables remained in the model, and absence of coronary artery disease was added as another predictive variable (Table 5). Regarding PR and AH interval, DEX group assignment and older age were predictive for both in univariable analysis, as well as hypertension and larger LAVI for PR interval (Table 4). In multivariable analysis, dexmedetomidine group assignment and age remained in both models, with LAVI added to predict PR interval and LVEF for prediction of AH interval (Table 5). Group assignment to dexmedetomidine and LAVI were also correlated to the Wenckebach cycle length of the AV node in univariable analysis (Table 4). In multivariable analysis, hypertension and betablocker use were added as explaining variables (Table 5).

**Table 4:** Correlation of patient characteristics with sinus rate (RR interval) and AV conduction measurements during sedation

	**RR interval**	***P* value**	**PR interval**	***P* value**	**AH interval**	***P* value**	**Wenckebach CL of AVN**	***P* value**
**Variable**	**Coeff. (95%-CI)**		**Coeff. (95%-CI)**		**Coeff. (95%-CI)**		**Coeff. (95%-CI)**	
Group (Pro—Dex)	-116 (-191 to -41)	0.003	-20 (-33 to -8)	0.002	-15 (-26 to -4)	0.008	-56 (-95 to -17)	0.006
Age	5.80 (2.64 to 8.96)	< 0.001	1.04 (0.53 to 1.56)	< 0.001	0.62 (0.07 to 1.17)	0.027	1.63 (-0.11 to 3.36)	0.065
Gender	-28.5 (-105.6 to 48.6)	0.466	9.5 (-4.2 to 23.3)	0.172	9.0 (-4.3 to 22.4)	0.183	7.7 (-33.2 to 48.6)	0.710
Hypertension	20.7 (-56.7 to 98.1)	0.598	14.8 (2.1 to 27.4)	0.023	9.6 (-1.6 to 20.8)	0.094	33.1 (-6.0 to 72.1)	0.096
Diabetes	-32.4 (-172.1 to 07.3)	0.648	7.6 (-14.9 to 30.1)	0.506	-2.5 (-18.7 to 13.6)	0.759	-27.4 (-83.0 to 28.2)	0.332
CAD	-86.2 (-193.0 to 20.5)	0.113	-1.9 (-19.9 to 16.2)	0.839	7.3 (-8.9 to 23.5)	0.376	17.8 (-30.3 to 65.8)	0.465
Hx of CHF	-60.7 (-182.0 to 60.7)	0.325	18.6 (-5.2 to 42.5)	0.125	11.4 (-7.6 to 30.4)	0.238	16.9 (-50.6 to 84.4)	0.622
OSAS	-44.4 (-162.9 to 74.2)	0.461	-0.7 (-20.0 to 18.7)	0.947	0.6 (-12.0 to 13.3	0.925	37.3 (-50.6 to 125.2)	0.403
LVEF	1.35 (-2.68 to 5.38)	0.508	0.37 (-0.50 to 1.24)	0.404	0.47 (-0.21 to 1.14)	0.173	0.91 (-1.76 to 3.58)	0.501
LAVI	3.94 (0.80 to 7.09)	0.014	0.73 (0.17 to 1.28)	0.010	0.35 (-0.20 to 0.90)	0.208	2.18 (0.40 to 3.96)	0.017
Betablocker	23.4 (-59.4 to 106.2)	0.577	2.2 (-11.3 to 15.7)	0.744	-6.3 (-18.5 to 5.8)	0.305	-40.4 (-82.4 to 1.6)	0.059
Procedure duration	-0.18 (-0.77 to 0.42)	0.557	-0.01 (-0.11 to 0.10)	0.915	-0.04 (-0.13 to 0.05)	0.385	-0.18 (-0.48 to 0.13)	0.254

*AVN* atrioventricular node, *CAD* coronary artery disease, *CHF* congestive heart failure, *CI* confidence interval, *CL* cycle length, *Coeff* coefficient, *DEX* dexmedetomidine, *Hx* history, *LAVI* left ventricular volume index, *LVEF* left ventricular ejection fraction, *OSAS* obstructive sleep apnea syndrome, *PRO* propofol

**Table 5:** Multivariable correlation of patient characteristics with sinus rate (RR interval) and AV conduction measurements during sedation

	**RR interval**	***P*-value**	**PR interval**	***P*-value**	**AH interval**	***P*-value**	**Wenckebach CL of AVN**	***P*-value**
**Variables**	**Coeff. (95%-CI)**		**Coeff. (95%-CI)**		**Coeff. (95%-CI)**		**Coeff. (95%-CI)**	
Group (Pro—DEX)	-110.7 (-181.9 to -39.4)	0.003	-17.7 (-29.7 to -5.7)	0.004	-15.9 (-27.1 to -4.7)	0.006	-46.2 (-83.1 to -9.2)	0.015
Age^a^	5.26 (2.10 to 8.41)	0.001	0.90 (0.43 to 1.36)	< 0.001	0.57 (0.06 to 1.09)	0.030	-	-
CAD	-118.9 (-243.3 to 5.4)	0.061	-	-	-	-	-	-
LAVI^a^	3.52 (0.37 to 6.67)	0.029	0.52 (-0.01 to 1.06)	0.054	-	-	2.08 (0.19 to 3.97)	0.031
LVEF	-	-	-	-	0.55 (-0.14 to 1.24)	0.115	-	-
Hypertension	-	-	-	-	-	-	34.3 (-1.1 to 69.6)	0.058
Betablocker	-	-	-	-	-	-	-47.9 (-85.3 to -10.4)	0.013

*AVN* atrioventricular node, *CAD* coronary artery disease, *CI* confidence interval, *CL* cycle length, *Coeff* coefficient, *LAVI* left ventricular volume index, *DEX* dexmedetomidine, *LVEF* left ventricular ejection fraction, *PRO* propofol

^a^for 1 unit increase

## Discussion

Deep sedation with dexmedetomidine, in comparison to propofol, affects suprahissian conduction. It results in prolongation of the PR and AH interval and increases the Wenckebach cycle length of the atrioventricular node. Dexmedetomidine also lowers sinus rate compared to propofol, with prolongation of SNRT, but both corrected as well as normalized SNRT are not different.

Bradycardia is a well-described phenomenon of dexmedetomidine sedation. Cases of cardiac arrest of up to 4 min duration have been described during dexmedetomidine infusion, necessitating cardiopulmonary resuscitation.[12, 13] Several meta-analysis confirmed an increased risk of bradycardia during dexmedetomidine sedation.[14] Cases of prolonged, complete AV block have also been reported with dexmedetomidine sedation, and age > 50 years and cardiac comorbidities described as risk factors.[15, 16] Likewise, the occurrence of complete AV block has been reported during propofol administration,[17–19] and propofol infusion does also increase the risk of bradycardia.[20].

So far, no head-to-head comparison of the electrophysiological effects of dexmedetomidine versus propofol has been performed. However, previous electrophysiological studies showed that dexmedetomidine has an impact on both AV and sinus node function. Sairaku randomized 215 patients to receive or not to receive dexmedetomidine during an electrophysiological study. They reported a longer, corrected SNRT, Wenckebach cycle length of the AV node, AV nodal ERP and AH interval in patients with dexmedetomidine sedation.[21] Poyhia performed an electrophysiological study in 11 patients before and after dexmedetomidine infusion.[22] They found a prolonged cSNRT, higher Wenckebach cycle length of the AV node and ERP of the AV node. Sinus cycle length and SNRT were not different in their study. Ergul et al. showed in 20 children that dexmedetomidine increased sinus rate and prolonged SNRT, cSNRT, AV Wenckebach cycle length of the AV node and AV nodal ERP.[23] However, they found no effect of dexmedetomidine on AH interval.

Previous electrophysiological studies on the effect of propofol on sinus and AV node function show a less consistent pattern. In a cross-over study, Warpechowski investigated the effect of propofol on AV nodal conduction properties in 12 patients with AV nodal reentry tachycardia. [24] They found no effect of propofol on AH and HV interval and on ERP of both the fast (antegrade and retrograde) and slow (antegrade) pathways. Similarly, Sharpe et al. demonstrated in 12 patients with Wolff-Parkinson-White syndrome that propofol, compared to alfentanyl and midazolam, did not affect the ERP of the AV node and sinus node function. In a pig model, propofol was shown to decrease sinus rate, to prolong cSNRT and to increase the HV interval in a dose-dependent way.[25] Matsushima recently reported that high-dose propofol in 23 pediatric patients prolonged the HV interval, but was without effect on AH interval and SNRT.[26].

Our randomized controlled study, directly comparing the electrophysiological effects of both drugs, clearly confirms a more pronounced effect of dexmedetomidine on sinus rate as well as on suprahissian conduction of the AV node. Age was the most important modifying factor besides group allocation regarding both sinus rate, PR and AH interval. Also importantly, we did not find a difference of the two drugs on infrahissian conduction, as both HV interval and QRS were not different among groups. Likewise, after correction of heart rate, we did not observe any difference in ventricular repolarization between the two sedatives. Our findings have clinical implications. Although both drugs have proven safe for deep sedation in various settings, propofol may be preferred over dexmedetomidine for deep sedation in patients with sinus bradycardia or AV conduction abnormalities. In particular in clinical settings, in which emergency cardiac pacing is not available.

Arrhythmia inducibility is another important point to consider during electrophysiological studies. To this regard it is reassuring that we did not find any difference in arrhythmia inducibility among groups. Similarly, in the study by Sairaku et al., atrial fibrillation inducibility was not different among patients with versus without dexmedetomidine administration.[21] Previous studies already reported that inducibility of supraventricular tachycardia is not compromised by both drugs. In 326 patients undergoing electrophysiological study for paroxysmal, supraventricular tachycardia, arrhythmia inducibility was retrospectively investigated by Slupe et al.[27] Compared to fentanyl and midazolam alone, the addition of dexmedetomidine did not affect arrhythmia inducibility. In the study by Matsushima described above, sustained reciprocating tachycardia were inducible in 8 of 12 patients, and propofol had no effect on electrophysiological properties.[26] However, because of the effect of dexmedetomidine on suprahissian conduction, propofol sedation may be preferred over dexmedetomidine sedation in patients with AV nodal reentry tachycardia. Our study is limited by the small number of patients and the results cannot be generalized to all patients, as patients with advanced conduction abnormalities or bradycardia and patients with impaired left ventricular function were excluded. Some DEX group patients had additional propofol administration for electrical cardioversion during the procedure, which may have affected the results of the EP study. However, the median dose of propofol administered was only 20 mg and the EP study was performed at least 10–15 min after propofol administration.

## Conclusions

Dexmedetomidine, compared to propofol, has a more pronounced effect on sinus rate and suprahissian conduction of the AV node. No differences among the two drugs were observed on infrahissian conduction and ventricular repolarization. In patients with sinus or AV node disease propofol may be preferred over dexmedetomidine for deep sedation.

## Data Availability

The datasets used and/or analysed during the current study are available from the corresponding author on reasonable request.

## Abbreviations

AH: atrial - His
HV: His - ventricular
AV: atrioventricular
CL: cycle length
cSNRT: corrected sinus node recovery time
ERP: effective refractory period
nSNRT: normalized sinus node recovery time
SCL: sinus cycle length
SNRT: sinus node recovery time
MOAA/S: modified observer’s assessment of alertness/sedation
TCI: targedt controlled infusion
